# Measuring the unmeasurable multidimensional poverty for economic development: Datasets, algorithms, and models from the poorest region of Luzon, Philippines

**DOI:** 10.1016/j.dib.2024.110150

**Published:** 2024-02-05

**Authors:** Emmanuel A. Onsay, Jomar F. Rabajante

**Affiliations:** a Graduate School, University of the Philippines Los Baños, Laguna 4030, Philippines; b Partido Institute of Economics, Partido State University, Camarines Sur 4422, Philippines

**Keywords:** Rural poverty, Calamity occurrences, Disaster risk preparedness, Advanced econometrics, Data analytics, Regression, Policy, Philippines

## Abstract

Poverty is the oldest social problem that ever existed and is difficult to reverse. It is multidimensional and unmeasurable. Thus, measuring by decomposing rural multidimensional poverty is critical. Most poverty studies are usually generic, exposed to large sampling errors, and intended for macroeconomic decisions. Thus, measuring poverty for a specific locality with various configurations is crucial for economic development. This work presents a processed and analyzed dataset from a huge community-based monitoring system of Goa, Camarines Sur. The local is situated in the poorest district, of the poorest province, in the poorest region of Luzon, Philippines. Research about poverty in this area is limited and measuring poverty at specific locality is scarce. The datasets contain the multidimensional poverty indicators, health, and nutrition, housing and settlement, water and sanitation, basic education from elementary to senior high school, income classifications, employment and livelihood, peace and order, summary of calamity occurrences experienced by residents, disaster risk reduction preparedness, figures of diagnostic analytics, tables of descriptive analytics, poverty analytics, measurement of decomposed poverty, summary of disaggregated configurations, graphs of predictive and prescriptive analytics, and population dynamics. This work is vital in analyzing poverty in rural and multidimensional approaches through poverty incidence, poverty gap, severity statistics, watts index, and classifications. It may also serve as a basis for measuring poverty from nearby regions and nations that use complete enumeration of its households and members. By utilizing the analyzed and processed data, further classifications and regressions can be done. It can be freely used by the government, private organizations, charitable institutions, businesses, academia, and researchers to target policies. An advantage of utilizing the dataset is to address multifaceted poverty that requires different interventions. It will facilitate the creation of programs to alleviate poverty and promote local economic development.

Specifications Table

**Table:** 

Subject	Economic development and growth, econometrics, rural studies
Specific subject area	Rural studies, poverty economics, development studies, development economics, applications of data analytics on society, social studies, poverty econometrics, policy study
Data format	Raw Analyzed Filtered Processed
Type of data	Table, Chart, Graph, Figure
Data collection	The dataset comprised 36 indicators from 6 multidimensional poverty classifications of 4 sectors divided into 34 barangays at magnitude and proportion measurements of the municipality [1]. It was cleaned, filtered, transformed, coded, analysed, and processed from the huge community-based monitoring system that contained many variables that are not relevant for policy-making. The data collection was performed by the local government unit of Goa, Camarines Sur. A census (survey) of all household's population was conducted. The survey was made among the public to be able to address various problems of the residents. The researchers utilized various software such as R, STATA, Python, SPSS, and MS Excel to effectively and efficiently generate necessary data for economic development. Apart from it, descriptive, diagnostic, predictive, and prescriptive models were employed to derive useful data and analyzed data that will serve as input for economic development.
Data source location	The data were collected from the community-based monitoring system 2018-2020. The data is collected by the Local Government Unit of Goa, Camarines Sur, Philippines and is open for public usage, especially for the academia that served as their partners in data analysis and policy-making. The local government unit owns the data from the community-based monitoring system. The survey began in 2018 and the data validation was completed in 2020. The data was cross-sectional and covered the years 2018–2020. In the social sciences and economics, gathering and analyzing data is typically costly, time-consuming, and infamously complex. Through the collection of data for LGU utilization, the CBMS serves as a method for assessing poverty at the local level and improving accountability, transparency, and resource allocation. According to RA 11315, it can be utilized as a guide for developing successful programs and policies that promote community development while lowering poverty and identifying the underlying causes of poverty [13]. There are 14,021 Households with
	63,749 members that are being evaluated. Four groups were created, each of which served as the base for the multisectoral analysis [1]. The four sectors were classified using the municipality's sectoral classification system. The researchers served as the analysts and statisticians of the data to derive insights, create predictive summaries, perform advanced econometrics, and provide policies for economic development. The Partido State University and University of the Philippines Los Baños are authorized to utilize and analyze the datasets and disseminate its immediate summaries and findings for the socio-economic advancement of Filipinos.
Data accessibility	Repository name: Mendeley Data Data identification number: 10.17632/s76nh7dm4v.1 Direct URL to data: https://data.mendeley.com/datasets/s76nh7dm4v/1 Onsay, Emmanuel; Rabajante, Jomar (2023), “Dataset on Measuring the Unmeasurable Multidimensional Rural Poverty for Economic Development: Analysis from the Poorest District of the Poorest Province in the Poorest Region of Luzon, Philippines”, Mendeley Data, V1, doi: 10.17632/s76nh7dm4v.1

## 1 Value of the Data


 

• Measuring multidimensional poverty is difficult and challenging thus it is deemed unmeasurable, especially for rural areas and specific localities. The dataset is useful for providing information in designing programs and policies for poverty alleviation and economic development that are well-targeted for the poorest regions.
• The datasets will provide baseline data and indicators of multidimensional poverty for other developing countries and poor regions of the world to devise policies for economic development. Future researchers may adopt the data analytics procedures, variables used, methods employed, policy proposals, and computational styles to create similar studies on measuring the unmeasurable aspects of multidimensional poverty for other poor regions.
• The poverty analytics dataset can be used to compute, verify, and simulate poverty incidence, poverty gap, severity statistics, watts index, and classifications for various sectors and locals of poor regions.
• The processed datasets provide descriptive analytics and diagnostic analytics that measure population dynamics, health and nutrition, housing and settlement, water and sanitation, basic education from elementary to senior high school, income classifications, employment and livelihood, peace, and order, summary of calamity occurrences experienced by residents, disaster risk reduction preparedness.
• Local government units, National government agencies, researchers, scholars, extensionists, policy-makers, academicians, charitable institutions, and social enterprises will find the prescriptive analytics dataset useful in designing programs and monitoring impacts for socio-economic advancement, not just in the Philippines but in the rest of the world.
• The predictive analytics dataset and econometric models could provide empirical shreds of evidence to assert poverty theories on rural studies that will add literature on scarce multidimensional poverty measurements in the Philippines and other poor countries.

## 2 Background

Since time immemorial, poverty has been one of the most persistent social problems that ever occurred and has not yet been fully resolved. Haughton and Khandker (2009) claim that it has a negative effect on the growth of society and economic prosperity [2]. It is difficult to handle and revert. Numerous economists have tried to explain the causes of global poverty and the required changes. Nonetheless, assessing poverty in rural areas is more challenging, and there is a dearth of published research on the subject. Thus, researchers have been compiling necessary datasets from immense community-based monitoring systems to measure multidimensional poverty in their region to be able to come up with sound policy interventions that can alleviate poverty and promote economic development. Measuring the unmeasurable in the field of natural sciences has been conducted by various researchers [3,^4^,^7^,11]. However, Economic issues and social events are difficult to quantify, challenging to analyze, and some are unmeasurable [10]. A limited amount of research has also been done on the multifaceted nature of poverty in rural areas of the Philippines, the decentralization of poverty cases, economic progress, and sociological advancement, particularly in the Bicol region, is insufficient. Moreover, only a few studies have made use of complete enumeration techniques in examining the depths of poverty. In the Partido district, no research has been done to quantify poverty using predictive analytics and advanced econometrics. Bicol region is the poorest region in Luzon and Camarines Sur is the poorest province in the Bicol region with a poverty incidence of 38.7% [6]. Thus, the dataset is vital in addressing multidimensional poverty and promoting economic growth, not just in this locale but in the rest of the world by utilizing the models and indicators used in this work.

## 3 Data Description

Poverty is a substantial absence of well-being [2]. It has no standard definition because poverty is notoriously difficult to measure. Different countries or regions have varying definitions of poverty and its definition is based on the poverty line or food thresholds. Countries or regions around the globe have different poverty lines (z) or food thresholds (f). Thus, it is important to note that modeling poverty depends on a certain region or locality. For instance, in the Philippines, a household is deemed poor if they are earning PhP10,481 and below (around 186.18 USD) based on 2019-year statistics. Considering the foregoing, the datasets are very useful in understanding and modeling poverty, not just for a specific locale of the work but may serve as a benchmark in measuring poverty around the globe. The constructs and indicators we are proving and providing with this work are generally reproducible by scholars across regions.

When modeling and analyzing poverty using multidimensional parameters, these variable sets are quite helpful. The community-based monitoring system (CBMS) was mined, wrangled, and clustered; an all-in system was used to fit the model; bidirectional, forward selection, and backward elimination approaches were used; variables were categorized and the work was reviewed; the local government provided a benchmark; the physical observation of poverty through in-person visits was conducted; and data transparency and availability were taken into consideration. The poverty models presented in this work are intended to describe, diagnose, and predict multidimensional poverty through multiple constructs and indicators. By utilizing the datasets and generating useful statistics and analytics, a prescription to alleviate poverty and promote economic development could be provided.

The dataset in the repository contains 6 components, namely: the multidimensional poverty variables, the population dynamics, the descriptive analytics, the diagnostic analytics, the poverty analytics, the predictive policy, and the prescriptive analytics [1]. The multidimensional poverty variables are contained in an excel file with 34 indicators at household magnitude (21) and proportion measurements (15) of 4 sectors (Isarog, Poblacion, Ranggas, and Salog) of 34 Barangays.

Table 1 reveals 31 variables at magnitude measurements with corresponding descriptions and definitions. These sets of variables are very useful in multidimensional poverty modeling and analysis with multidimensional constructs. The indicators were chosen based on the clustering, mining, and wrangling of the community-based monitoring system (CBMS), model fitting through an all-in system, bidirectional, forward selection and backward elimination approaches, regression, and classification of authors, review of related work, benchmark from local government, physical observation of poverty through personal visitations, and data availability and transparency.

**Table 1:** Overview of data indicator characteristics at magnitude measurements.

VAR	Descriptions	Definitions
1. HHM	Household Magnitude	Frequency count at household magnitude for all barangays and sectors
2. MEMM	Membership Magnitude	Frequency count at membership magnitude for all barangays and sectors
3. α	Children under 5 years old who died	1 (HH with Children under 5 who died), 0 (HH without Children under 5 who died)
4. Ξ	Total HH with children under 5 years old	Number of households with children under 5 years old
5. ξ	Total population of children under 5 years old	Total population of children under 5 years old from HH
6. β	Women who died due to pregnancy related causes	1 (HH with Women who died due to pregnancy related cases), 0 (HH without Women who died due to pregnancy related cases)
7. γ	Malnourished children 0-5 years old	1 (HH with children aged 0-5 who are malnourished), 0 (HH without children aged 0-5 who are malnourished)
8. Ι	Total number of HH with children 0-5 years old	Total number of households with children 0-5 years old
9. ι	Total population of children aged 0-5 years old	Total population of children aged 0-5 years old from HH
10. δ	Households living in makeshift housing	1 (HH who are living in Makeshift Housing), 0 (HH who are not living in Makeshift Housing)
11. ε	Households who are informal settlers	1 (HH who are informal settlers), 0 (HH who are not living in Makeshift Housing)
12. ζ	Households without access to safe water	1 (HH without Access to Safe Drinking Water), 0 (HH with Access to Safe Drinking Water)
13. η	Households without access to sanitary toilet facility	1 (HH without Access to Sanitary Toilet Facility), 0 (HH with Access to Sanitary Toilet Facility)
14. θ	Children aged 6-11 years old who are not attending elementary	1 (HH with children not attending elementary), 0 (HH with children attending elementary)
15. Κ	Total # of HH with children aged 6-11	Total number of HH with children aged 6-11 years old (Qualified for elementary level)
16. κ	Total population of children aged 6-11 years old	Total population of children aged 6-11 years old (Qualified for elementary level) from HH
17. λ	Children aged 12-15 years old who are not attending Junior High School	1 (HH with children not attending junior high school), 0 (HH with children attending junior high school)
18. Ο	Total # of HH with children aged 12-15 years old	Total number of HH with children aged 12-15 years old (Qualified for junior high school level)
19. ο	Total population of children aged 12-15 years old	Total population of children aged 12-15 years old (Qualified for junior high school level) from HH
20. µ	Children aged 16-17 years old not attending Senior High School	1 (HH with children not attending senior high school), 0 (HH with children attending senior high school)
21. π	Total # of HH with children aged 16-17	Total number of HH with children aged 16-17 years old (Qualified for senior high school level)
22. Π	Total population of children aged 16-17	Total population of children aged 16-17 years old (Qualified for senior high school level) from HH
23. ρ	Households with income below poverty threshold	1 (HH Living below Poverty Threshold), 0 (HH Not Living below Poverty Threshold)
24. φ	Households with income below food threshold	1 (HH Living below Food Threshold), 0 (HH Not Living below Food Threshold)
25. χ	Households who experienced food shortage	1 (HH with food shortage), 0 (HH without food shortage)
26. ψ	Unemployed members of the labor force	1 (HH with unemployment), 0 (HH without unemployment)
27. Υ	*Total # of HH with members of the labor force	Total number of HH with members of labor force (active)
28. υ	*Total population of members of the labor force	Total population of labor force (active) from HH
29. ω	Victims of crime	1 (HH with victims of crime), 0 (HH without victims of crime)
30. H	Total Number of Households	Frequency count of all households
31. N	Total Population	Frequency count of all household members

The abovementioned table depicts 17 variables at proportion measurements that can be utilized for descriptive, diagnostics, predictive, and prescriptive modeling and analysis of poverty at various configurations. They were selected based on the core poverty indicators of the community-based monitoring system (CBMS), empirical shreds of evidence of causations from various models of authors, review of related literature, a benchmark from local government unit, physical observation of faces of poverty, and data availability and transparency.

Table 3 summarizes the 17 variables with the 7 main poverty clusters at magnitude and proportion measurements. These clusters are vital for poverty characterization and visualization purposes.

**Table 2:** Overview of data indicator characteristics at proportion measurements.

Variables	Descriptions	Definitions
32. HHP	Households Proportion	Proportions from household magnitude for all barangays and sectors
33. MEMP	Members Proportion	Proportions from membership magnitude for all barangays and sectors
34. Α	Children under 5 years old who died	1 (HH with Children under 5 who died), 0 (HH without Children under 5 who died)
35. Β	Women who died due to pregnancy related causes	1 (HH with Women who died due to pregnancy related cases), 0 (HH without Women who died due to pregnancy related cases)
36. Γ	Malnourished children 0-5 years old	1 (HH with children aged 0-5 who are malnourished), 0 (HH without children aged 0-5 who are malnourished)
37. Δ	Households living in makeshift housing	1 (HH who are living in Makeshift Housing), 0 (HH who are not living in Makeshift Housing)
38. Ε	Households who are informal settlers	1 (HH who are informal settlers), 0 (HH who are not living in Makeshift Housing)
39. Ζ	Households without access to safe water	1 (HH without Access to Safe Drinking Water), 0 (HH with Access to Safe Drinking Water)
40. S	Households without access to sanitary toilet facility	1 (HH without Access to Sanitary Toilet Facility), 0 (HH with Access to Sanitary Toilet Facility)
41. Θ	Children aged 6-11 years old who are not attending elementary	1 (HH with children not attending elementary), 0 (HH with children attending elementary)
42. Λ	Children aged 12-15 years old who are not attending Junior High School	1 (HH with children not attending junior high school), 0 (HH with children attending junior high school)
43. Μ	Children aged 16-17 years old not attending Senior High School	1 (HH with children not attending senior high school), 0 (HH with children attending senior high school)
44. Ρ	Households with income below poverty threshold	1 (HH Living below Poverty Threshold), 0 (HH Not Living below Poverty Threshold)
45. Φ	Households with income below food threshold	1 (HH Living below Food Threshold), 0 (HH Not Living below Food Threshold)
46. Χ	Households who experienced food shortage	1 (HH with food shortage), 0 (HH without food shortage)
47. Ψ	Unemployed members of the labor force	1 (HH with unemployment), 0 (HH without unemployment)
48. Ω	Victims of crime	1 (HH with victims of crime), 0 (HH without victims of crime)

**Table 3:** Multidimensional clusters of variables that can be used for poverty modeling and analysis at magnitude and proportion measurements.

Variables	Underlying Poverty Cluster	Descriptions
49. 诶	Health and Nutrition	Children under 5 years old who died
		Total HH with children under 5 years old
		Total population of children under 5 years old
		Women who died due to pregnancy related causes
		Malnourished children 0-5 years old
		Total number of HH with children 0-5 years old
		Total population of children aged 0-5 years old
50. 必	Housing and Settlement	Households living in makeshift housing
		Households who are informal settlers
51. 西	Water and Sanitation	Households without access to safe water
		Households without access to sanitary toilet facility
52. 弟	Basic Education	Children aged 6-11 years old who are not attending elementary
		Total # of HH with children aged 6-11
		Total population of children aged 6-11 years old
		Children aged 12-15 years old who are not attending Junior High School
		Total # of HH with children aged 12-15 years old
		Total population of children aged 12-15 years old
		Children aged 16-17 years old not attending Senior High School
		Total # of HH with children aged 16-17
		Total population of children aged 16-17
53. 衣	Income and Livelihood	Households with income below poverty threshold
		Households with income below food threshold
		Households who experienced food shortage
54. 艾付	Employment	Unemployed members of the labor force
		*Total # of HH with members of the labor force
		*Total population of members of the labor force
55. 记	Peace and Order	Victims of crime
56. 爱耻	Multidimensional Poverty Indicators	Measurements of Multidimensional Poverty Indicators
57. 挨	Households	Measurements of Multidimensional Poverty Indicators at Households
58. 宅	Members	Measurements of Multidimensional Poverty Indicators at Members

Table 4 lists the 4 primary sectors of the municipality. The four sectors were classified using the municipality's sectoral classification system. The Isarog sector was coined from the tallest forested peak on Southern Luzon, the Mt. Isarog. It is also known as the Upland Sector, named for the fact that 12 of its barangays are situated on Mt. Isarog's slope. The Poblacion sector serves as the commercial and economic hub and consists of 10 barangays. While, the Bicol word “Riverine,” which describes towns near rivers, is the source of the word “Salog” sector. All barangays in the Salog Sector have access to several streams. The longest river in the area is called The Ranggas, and it runs from Mount Isarog to the seaside communities of Partido.

**Table 4:** The sectors and barangays of the municipality for population dynamics and poverty analytics.

Sectors		Barangays
Isarog	Abucayan	Payatan
	Balaynan	Pinaglabanan
	Digdigon	San Isidro West
	Hiwacloy	Scout Fuentebella
	Lamon	Tabgon
	Maysalay	Tamban
Poblacion	Bagumbayan Grande	San Benito
	Bagumbayan Pequeño	San Isidro
	Belen	San Jose
	La Purisima	San Juan Evangelista
	Panday	San Juan Bautista
Ranggas	Buyo	San Pedro
	Catagbacan	Tagongtong
	Matacla	
Salog	Cagaycay	Napawon
	Gimaga	Salog
	Halawigogon	Taytay
	Maymatan	

**Fig. 1:**
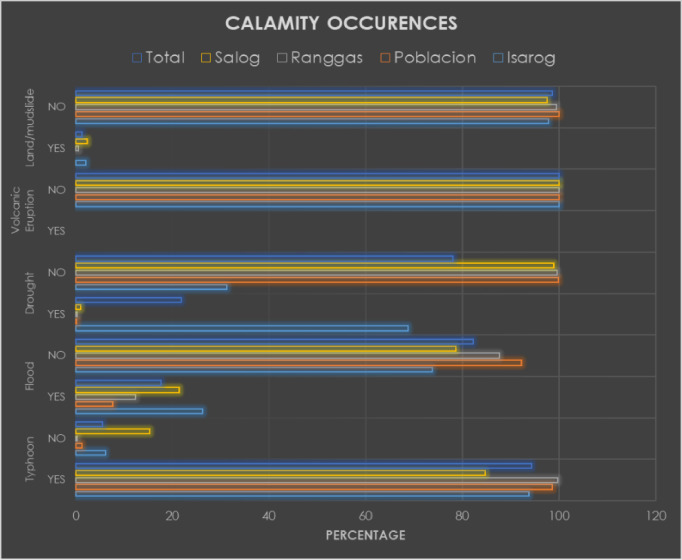
Multisectoral calamity occurrence analytics that can be used or reproduced by the public in other regions.

Fig. 1 shows an output of the dataset concerning the multisectoral calamity occurrences in all the sectors and municipalities. It was processed through descriptive analytics by summarizing all occurrences of natural disasters among 4 sectors. Based on the results, it is evident that the region is prone to natural hazards, particularly typhoons. The majority of all households suffer from the onslaught of typhoons since we are situated along the typhoon belt of the Philippines. Through effective data visualization, a large dataset was summarized into a figure that generates vital quick insights about the region.

**Fig. 2:**
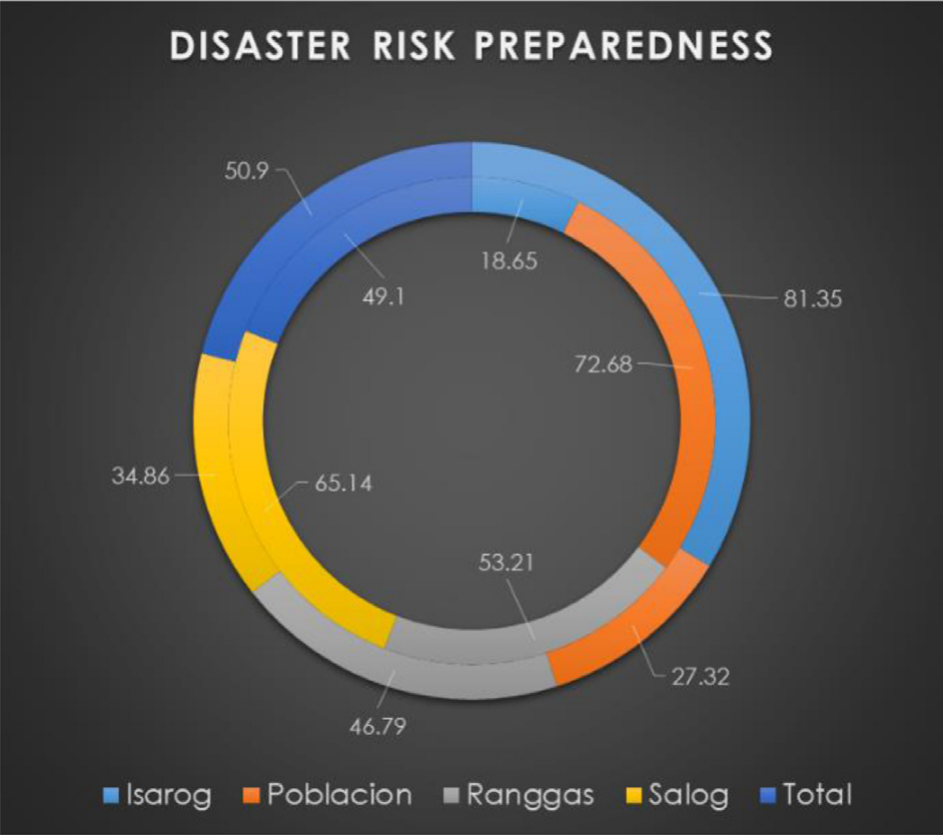
Multisectoral disaster risk preparedness analytics that can be used or reproduced by the public in other regions.

This is an output of diagnostic analytics where the disaster risk preparedness of households is analyzed and visualized (Fig. 2). More households in the Poblacion sector are prepared as compared with other sectors. By utilizing and producing this output, we can derive insights to analyze disaster risk preparedness in connection to calamity occurrence.

**Fig. 3:**
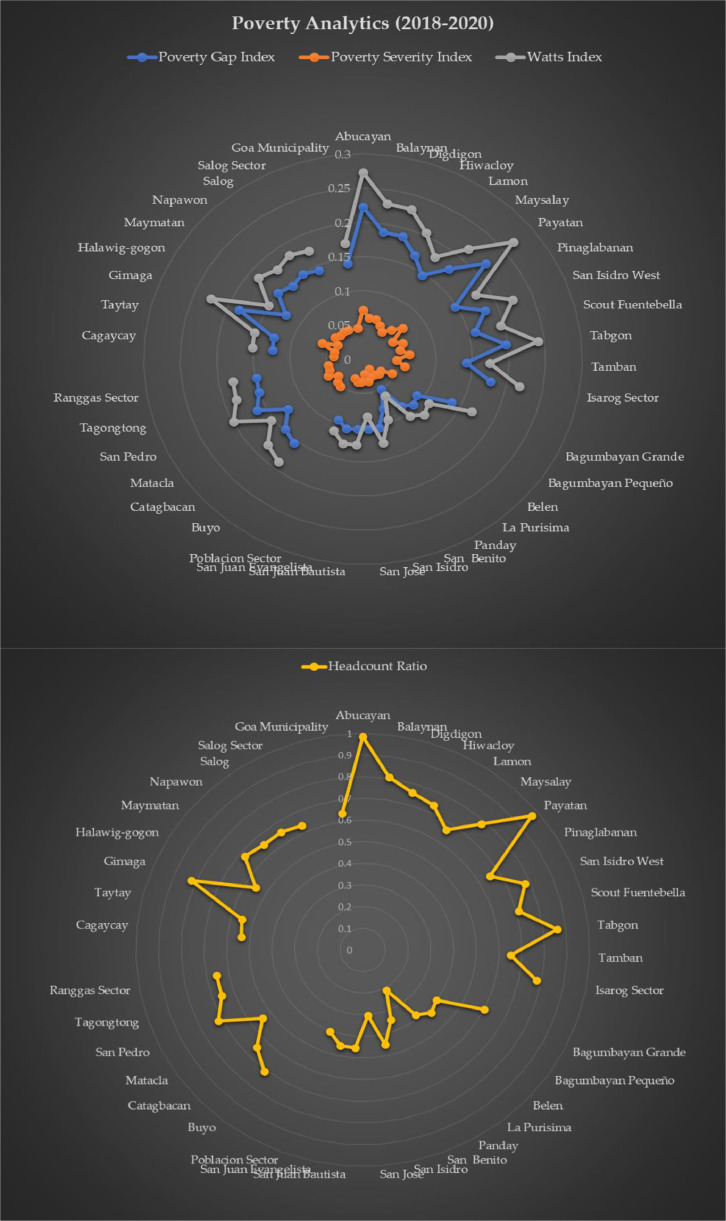
Poverty analytics at rural level that can be utilized or reproduced by the public in other regions.

Fig. 3 depicts the results of poverty analytics on the district and is being complemented by Table 5. 4 metrics were utilized, namely: headcount ratio which measures the incidence of poverty, gap ratio which measures the depth of poverty, severity ratio measures the intensity of poverty, and watts index that measures the degree of poverty at distributional functions. The poverty incidence is the proportion of the population that is comprised of those who live in poverty. The headcount ratio has the drawback of ignoring the degree of poverty; as the poor get poorer, the headcount index stays the same. Thus, the Poverty gap index is necessary. It is the percentage of the poverty line that the average income disparity in the population is expressed as. It assesses how far on average the impoverished fall below the poverty line to establish the level of poverty. To assess the intensity of poverty, the squared poverty gap index is derived. The statistic gives more weight to a poor person's observed income when it declines below the poverty line by squaring each data point on the poverty gap. It is the weighted sum of poverty gaps, whose weight is proportional to the size of the gap. Watts index decomposition was made to reflect the total distributional property of poverty.

**Table 5:** Results of poverty analytics on all the barangays and sectors of the municipality.

Barangay	Headcount Ratio	Poverty Gap Index	Poverty Severity Index	Watts Index
Abucayan	0.9845	0.2228	0.0723	0.2724
Balaynan	0.8058	0.1876	0.0610	0.2294
Digdigon	0.7582	0.1872	0.0607	0.2288
Hiwacloy	0.7363	0.1672	0.0544	0.2045
Lamon	0.6642	0.1463	0.0475	0.1789
Maysalay	0.7812	0.1763	0.0574	0.2157
Payatan	0.9680	0.2188	0.0710	0.2676
Pinaglabanan	0.6553	0.1478	0.0480	0.1807
San Isidro West	0.7775	0.1819	0.0592	0.2225
Scout Fuentebella	0.7092	0.1586	0.0520	0.1943
Tabgon	0.8608	0.1970	0.0641	0.2410
Tamban	0.6506	0.1420	0.0461	0.1736
*Isarog Sector*	*0.7793*	*0.1778*	*0.0578*	*0.2175*
Bagumbayan Grande	0.6007	0.1368	0.0445	0.1673
Bagumbayan Pequeño	0.3990	0.0908	0.0295	0.1110
Belen	0.4190	0.0955	0.0311	0.1168
La Purisima	0.3795	0.0862	0.0280	0.1054
Panday	0.2138	0.0502	0.0168	0.0618
San Benito	0.3464	0.0767	0.0281	0.0951
San Isidro	0.4476	0.1026	0.0334	0.1255
San Jose	0.3048	0.1026	0.0220	0.0838
San Juan Bautista	0.4549	0.1024	0.0336	0.1253
San Juan Evangelista	0.4536	0.1036	0.0339	0.1269
*Poblacion Sector*	*0.4019*	*0.0947*	*0.0301*	*0.1119*
Buyo	0.7093	0.1547	0.0504	0.1893
Catagbacan	0.6468	0.1472	0.0478	0.1800
Matacla	0.5432	0.1257	0.0411	0.1539
San Pedro	0.7156	0.1629	0.0530	0.1992
Tagongtong	0.6558	0.1497	0.0487	0.1831
*Ranggas Sector*	*0.6542*	*0.1480*	*0.0482*	*0.1811*
Cagaycay	0.5392	0.1240	0.0404	0.1517
Taytay	0.5507	0.1256	0.0409	0.1537
Gimaga	0.8220	0.1846	0.0601	0.2259
Halawig-gogon	0.5538	0.1235	0.0402	0.1511
Maymatan	0.6773	0.1518	0.0496	0.1859
Napawon	0.6531	0.1433	0.0465	0.1752
Salog	0.6508	0.1486	0.0483	0.1818
Salog Sector	0.6353	0.1431	0.0466	0.1750
*Goa Municipality*	*0.6370*	*0.1409*	*0.0457*	*0.1714*

**Fig. 4:**
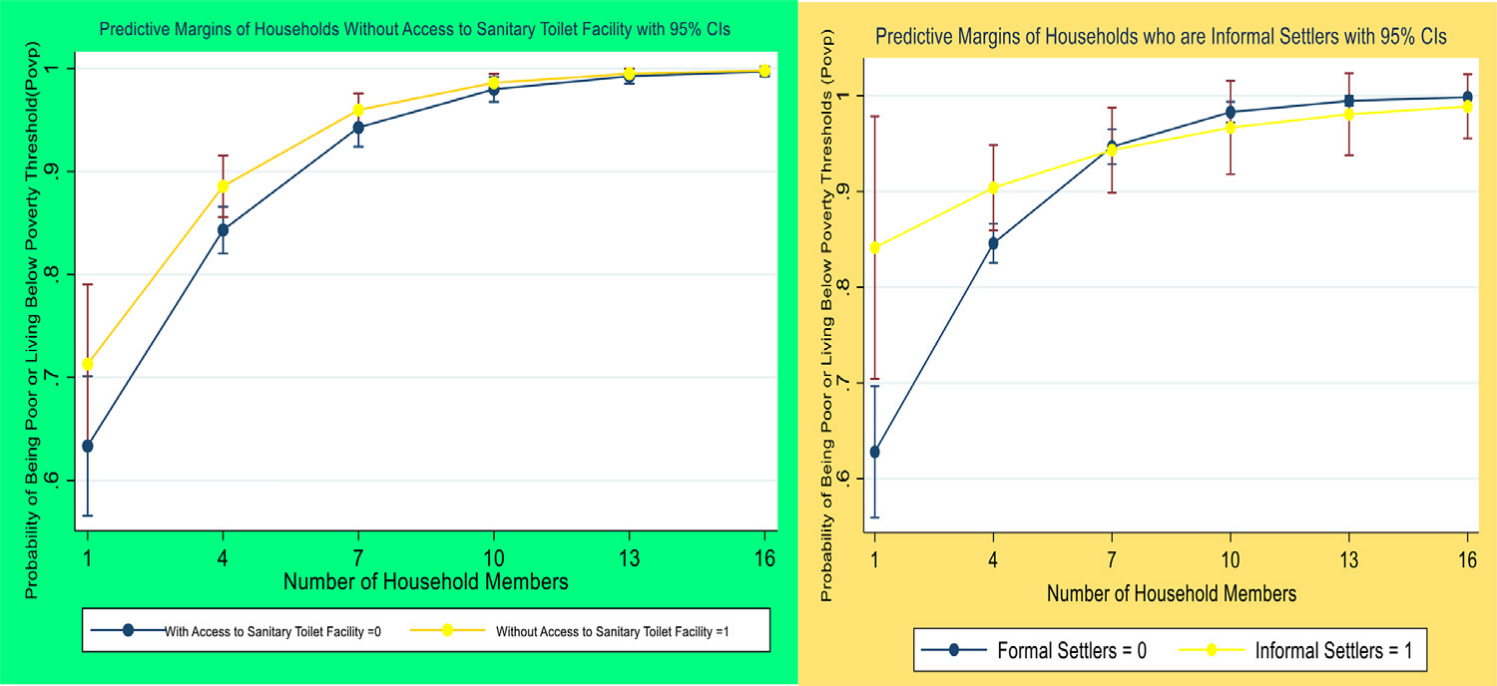
Classification results and predictive margins of multidimensional variables that can be used or reproduced by the public in other regions.

Fig. 4 illustrates the classification results from predictive analytics that analyzes the performance of the model. It shows the combination of interacting variables such as the number of households and access to sanitary toilet facilities, and informal settlement in connection to the probability of being poor or living below the poverty line. The graphs provide significant information on the empirical relationship and causation of variables that may be reconstructed using other variables or may be reproduced in other poor regions.

The researcher also employed a variety of interaction variables, as the image illustrates. The four factors that have an adverse effect on poverty outcomes are households without access to safe water x household members; households without access to safe water x informal settlers; households without access to safe water x households living in temporary housing; and households without access to a sanitary toilet facility x household living in temporary housing. It is clear that as housing, water, and sanitation indicators improve, the chance of a family becoming impoverished decreases. Informal settlers are more likely than formal settlers to experience poverty. Additionally, as the number of household members rises, so does the likelihood of becoming poor. The chance that a household will fall below the poverty line is lowest for households with fewer members and access to a sanitary restroom; it then rises for households with a large number of members and access to a sanitary restroom; finally, it falls for households without access to a sanitary restroom and fewer members. The findings demonstrate that one important factor influencing health dynamics is household size, which is a strong predictor of poverty. These results can be reused or duplicated by other scholars and researchers of poverty in poor regions of the world.

## 4 Experimental Design, Materials and Methods

The researchers generated, filtered, cleaned, and analyzed the dataset from the immense community-based monitoring system of the Local government unit of Goa, Camarines Sur from 2018–2020. Researchers utilized causal-explanatory design, predictive analytics, and advanced econometric models to generate the processed datasets. The researchers are authorized to process the dataset to be utilized for policy-making purposes. In data generation and analysis, the researchers used MS Excel, R, Stata, Python, and SPSS to disaggregate, decompose, and scrutinized the datasets.

The fundamental theoretical concepts or ideas that we attempt to quantify or examine are referred to as constructs. Based on economic theories or assumptions, we generate these abstract and frequently unobservable ideas. The multiple constructs in measuring multidimensional poverty are depicted through our models and analytics. The variables that are observable and measurable that are utilized as a stand-in for the constructs are called indicators. We employ these particular measures or data points to quantify or capture the relevant constructs.

Table 6 summarizes the multidimensional poverty indicators composed by the authors that can be utilized by the public for economic development and poverty alleviation efforts. There are 2 dependent variables, namely: poverty outcomes at the income (z) threshold (poverty based on a poverty line set by a specific region or country), and poverty outcomes at the food (f) threshold (poverty based on a food line set in specific region or country). 19 multidimensional poverty indicators may be utilized in the models as independent variables with 13 interacting variables. These indicators were chosen based on econometric modelling, clustering, benchmarks from related literature, data availability, and diagnostic analytics of the authors.

**Table 6:** Multidimensional poverty constructs and indicators composed by the authors that can be utilized by the public for economic development.

Variables		VAR	Description	A Priori Expectation *(Expected Signs of Coefficient)*
Dependent Variables	Poverty Outcomes at income (z)	*POVC_IT*	1 (Yes/Poor/HH Living below Poverty Threshold), 0 (No/Non-Poor/ HH Not Living below Poverty Threshold)	
	Poverty Outcomes at food (f)	*POVC_FT*	1 (Yes/Poor/HH Living below Food Threshold), 0 (No/Non-Poor/ HH Not Living below Food Threshold)	
Independent Variables	Child Mortality	*C_Mortality*	1 (HH with Children under 5 who died), 0 (HH without Children under 5 who died)	*Positive*
	Maternal Mortality	*M_Mortality*	1 (HH with Women who died due to pregnancy related cases), 0 (HH without Women who died due to pregnancy related cases)	*Positive*
	Malnutrition of Children	*C_Malnutrition*	1 (HH with children aged 0-5 who are malnourished), 0 (HH without children aged 0-5 who are malnourished)	*Positive*
	Type of Housing	*MSH_Dwellers*	1 (HH who are living in Makeshift Housing), 0 (HH who are not living in Makeshift Housing)	*Positive*
	Type of Settlement	*I_Settlers*	1 (HH who are informal settlers), 0 (HH who are not living in Makeshift Housing)	*Negative*
	Access to Safe Drinking Water	*WAS_Water*	1 (HH without Access to Safe Drinking Water), 0 (HH with Access to Safe Drinking Water)	*Positive*
	Access to Sanitary Toilet Facility	*WASF_Toilet*	1 (HH without Access to Sanitary Toilet Facility), 0 (HH with Access to Sanitary Toilet Facility)	*Positive*
	Total Number of Household Members	*TNOHH_Members*	The total number of members of Households	*Positive*
	Calamity Occurrence – Typhoon	*CO_Typhoon*	1 (HH who experienced typhoon), 0 (HH who did not experienced typhoon)	*Negative*
	Calamity Occurrence – Flood	*CO_Flood*	1 (HH who experienced flood), 0 (HH who did not experienced flood)	*Negative*
	Calamity Occurrence – Drought	*CO_Drought*	1 (HH who experienced drought), 0 (HH who did not experienced drought)	*Negative*
	Calamity Occurrence – Volcanic Eruption	*CO_VolEruption*	1 (HH who experienced volcanic erruption), 0 (HH who did not experienced volcanic eruption)	*Negative*
	Calamity Occurrence – Land/Mudslide	*CO_LMSlide*	1 (HH who experienced land/mudslide), 0 (HH who did not experienced land/mudslide)	*Negative*
	Disaster Risk Preparedness	*DR_Prepared*	1 (HH with disaster risk preparedness), 0 (HH without disaster risk preparedness)	*Negative*
	Children not attending Elementary	*CNA_Elem*	1 (HH with children not attending elementary), 0 (HH with children attending elementary)	*Positive*
	Children not attending Junior High School	*CNA_JunHS*	1 (HH with children not attending junior high school), 0 (HH with children attending junior high school)	*Positive*
	Children not attending Senior High School	*CNA_SenHS*	1 (HH with children not attending senior high school), 0 (HH with children attending senior high school)	*Positive*
	Unemployment	*LF_Unmployed*	1 (HH with unemployment), 0 (HH without unemployment)	*Positive*
	Victims of Crime	*Vic_Crime*	1 (HH with victims of crime), 0 (HH without victims of crime)	*Negative*

To determine the attributes of variables that influence a poverty status, a study may utilize the various models devised by Filipino economists who works on poverty economics and economic development, with modifications and multiple fittings [5,^8^,9]. The poverty statuses of households based on income and food thresholds are the dependent variables, while multidimensional variables, calamity occurrences, and disaster risk preparedness are the independent factors. In addition, the models included a number of control variables and intervening variables.

Y=α+Xβ+i+μ

Where: Y = logit (p) = log [p / (1- p)], p = probability of being poor of household or individual;

α = the intercept or individual effects of socio-economic conditions, education, health and nutrition, water and sanitation, housing and settlement, employment and livelihood, peace and order, calamity occurrences, and disaster risk preparedness which is assumed to be constant;

X = vector of independent variables or socio-economic conditions, education, health and nutrition, water and sanitation, housing and settlement, employment and livelihood, peace and order, calamity occurrences, and disaster risk preparedness, including control variables;

β = vector of coefficients, intercepts, or effects of socio-economic conditions, education, health and nutrition, water and sanitation, housing and settlement, employment and livelihood, peace and order, calamity occurrences, and disaster risk preparedness on poverty outcomes; i = intervening variables or combined effects of various socio-economic conditions, education, health and nutrition, water and sanitation, housing and settlement, employment and livelihood, peace and order, calamity occurrences, and disaster risk preparedness; and

μ = error term.

Logistic regression was employed to reveal the link of multidimensional variables on poverty outcomes. The Econometric Models below was used for predictive analytics. This is an econometric design that is concerned with establishing cause and effect between given variables with binary outcomes for rural setting. The logit models in this study were estimated as follows:

Model 1

$$\begin{array}{ccc} \text{POVC}\_\text{IT} & = & \beta_{0}+\beta_{1}C\_\text{Mortality}+\beta_{2}M\_\text{Mortality}+\beta_{3}C\_\text{Malnutrition}\\ & & +\;\beta_{4}\text{MSH}\_\text{Dwellers}+\beta_{5}I\_\text{Settlers}+\beta_{6}\text{WAS}\_\text{Water}+\beta_{7}\text{WASF}\_\text{Toilet}\\ & & +\;\beta_{8}\text{TNOHH}\_\text{Members}+\beta_{9}\text{CO}\_\text{Typhoon}+\beta_{10}\text{CO}\_\text{Flood}+\beta_{11}\text{CO}\_\text{Drought}\\ & & +\;\beta_{12}CO\_\text{VolEruption}+\beta_{13}\text{CO}\_\text{LMSlide}+\beta_{14}\text{DR}\_\text{Prepared}+\beta_{15}\text{CNA}\_\text{Elem}\\ & & +\;\beta_{16}\text{CNA}\_\text{JunHS}+\beta_{17}\text{CNA}\_\text{SenHS}+\beta_{18}\text{LF}\_\text{Unmployed}+\beta_{19}\text{Vic}\_\text{Crime}+\beta_{n}i+\mu \end{array}$$

Model 2

$$\begin{array}{ccc} \text{POVC}\_\text{FT} & = & \beta_{0}+\beta_{1}C\_\text{Mortality}+\beta_{2}M\_\text{Mortality}+\beta_{3}C\_\text{Malnutrition}\\ & & +\;\beta_{4}\text{MSH}\_\text{Dwellers}+\beta_{5}I\_\text{Settlers}+\beta_{6}\text{WAS}\_\text{Water}+\beta_{7}\text{WASF}\_\text{Toilet}\\ & & +\;\beta_{8}\text{TNOHH}\_\text{Members}+\beta_{9}\text{CO}\_\text{Typhoon}+\beta_{10}\text{CO}\_\text{Flood}+\beta_{11}\text{CO}\_\text{Drought}\\ & & +\;\beta_{12}CO\_\text{VolEruption}+\beta_{13}\text{CO}\_\text{LMSlide}+\beta_{14}\text{DR}\_\text{Prepared}+\beta_{15}\text{CNA}\_\text{Elem}\\ & & +\;\beta_{16}\text{CNA}\_\text{JunHS}+\beta_{17}\text{CNA}\_\text{SenHS}+\beta_{18}\text{LF}\_\text{Unmployed}+\beta_{19}\text{Vic}\_\text{Crime}+\beta_{n}i+\mu \end{array}$$

Poverty in multidimensional constructs may be measured by the abovementioned indicators. Poverty is multidimensional thus it must be captured by multidimensional variables. These variables include health and nutrition, housing and settlement, water and sanitation, basic education, livelihood and income, employment, natural disasters, disaster risk preparedness, and peace and order, with the presence of intervening variables. The model was designed by combining economic theories on microeconomics and developmental economics, mathematical theories, and statistical principles. Various indicators must be transformed into binary, categorical, and continuous outcomes to be used effectively. By following the data descriptors in Table 6, the proper coding of data may be achieved. The a-priori section depicts the expected sign of coefficients that is useful in poverty prediction. Positive coefficients are expected to increase poverty outcomes measured by income and food, while negative coefficients are expected to reduce the poverty outcomes measured by income and food. The model that we have constructed is a product of poverty econometrics that validates correlation and causation from immense data inputs through multidimensional constructs. By utilizing the indicators, the coefficients and odds ratios of independent variables may be measured which will provide significant insights into the probability of poverty outcomes in the municipality at ceteris paribus assumption. The aforementioned models may be modified to add or deduct indicators that can be used for predictive analytics, specific to a local or region.

To evaluate the extent of poverty the following measures may be utilized and generated. Onsay (2021) has generated the following measures and indices to analyze the rural poverty [5]:

Headcount Ratio. $P_{0}=\;\frac{1}{N}\;\sum_{i=1}^{N}\left(y_{i}<z\right)$; $P_{O}=\frac{N_{P}}{N}$ Where, Np = Number of poor; and N = Total Population (or sample). The headcount ratio (HCR) calculates the proportion of the population that is impoverished. When the expression included in brackets is true, the i function returns 1, and when it is false, it returns 0. A household is deemed poor if its income (yi) is less than the poverty line (z), in which case the i equals 1. The readability and simplicity of the headcount index are its main benefits. One drawback of the head count ratio is that it doesn't take into account the severity of poverty; as the poor get poorer, the headcount index doesn't change [2].

Poverty Gap Metrics. $P_{1}=\;\frac{1}{N}\;\sum_{i=1}^{N}\frac{G_{i}}{z}$ Where, $G_{i}$= (z - $x_{1})$ x $I(y_{i}<z)$. An indicator of how severe poverty is the poverty gap index. With the non-poor having none or no poverty gap, it is defined as the average poverty gap in the population represented as a percentage of the poverty line. By calculating the average distance below the poverty line, it establishes the level of poverty. The indicator is more closely aligned with zero when the proportion of the population living in poverty is lower and more closely aligned with one when that proportion is higher [2].

Poverty Severity. Squared Poverty Gap Index. $P_{\propto }=\;\frac{1}{N}\;\sum_{i=1}^{N}{\left(\frac{G_{i}}{z}\right)}^{\propto },\;\left(\propto \geq 0\right)$ Where ∝=sensitivity of index to poverty; z=poverty line; $x_{1}$=value of expenditure (income) per capita for ith person's HH; and $G_{i}$= z - $x_{1}$(with $G_{i}=0\;\text{when}\;x_{i\;}>z$) = poverty gap for individual i. The poverty gap index is correlated with the squared poverty gap index, sometimes referred to as the poverty severity index. The poverty gap ratio is multiplied by itself, and the average is then determined. The metric gives greater weight to an individual's observed income when it goes below the poverty level by squaring each data set representing the poverty gap. A weighted total of poverty gaps whose weight varies with gap size is the squared poverty gap index. It also takes poverty inequality into account [12].

Watts Index. $W=\;\frac{1}{N}\;\sum_{i=1}^{N}\left[\text{ln}\left(z\right)-\text{ln}\left(y_{i}\right)\right]=\left(\frac{1}{N}\right)\;\sum_{i=1}^{q}\text{ln}\left(\frac{z}{y_{i}}\right)$ Where the population's income (or spending) is indexed in ascending order for N individuals, and the total is divided by the number of individuals (q) whose income (yi) is below the poverty line (z). The poverty line is divided by income, logs are computed, the impoverished are added, and the index is then divided by the total population. This is one of the earliest measurements of poverty that takes distribution into account [2].

The input and output were categorized according to Estat classification. True denotes the binary outcomes of whether a household is poor or not. There are 1121 non-poor samples and 4648 poor samples in all. All 13,767 data are correctly classified by the model, demonstrating that sensitivity is 100%. The Specify is zero because none of the 225 observations could be conclusively classified as being below the poverty threshold. Thus, the model accurately predicted each and every home that was below the poverty threshold. The overall accuracy rate ranges from 73.29 to 74.12%. The models or alternative specifications used in the logistic model correctly classify the household observations. The same procedures may be used or reproduced by other researchers to gauge in poverty in their respective regions (Fig. 4, Table 8).

**Fig. 5:**
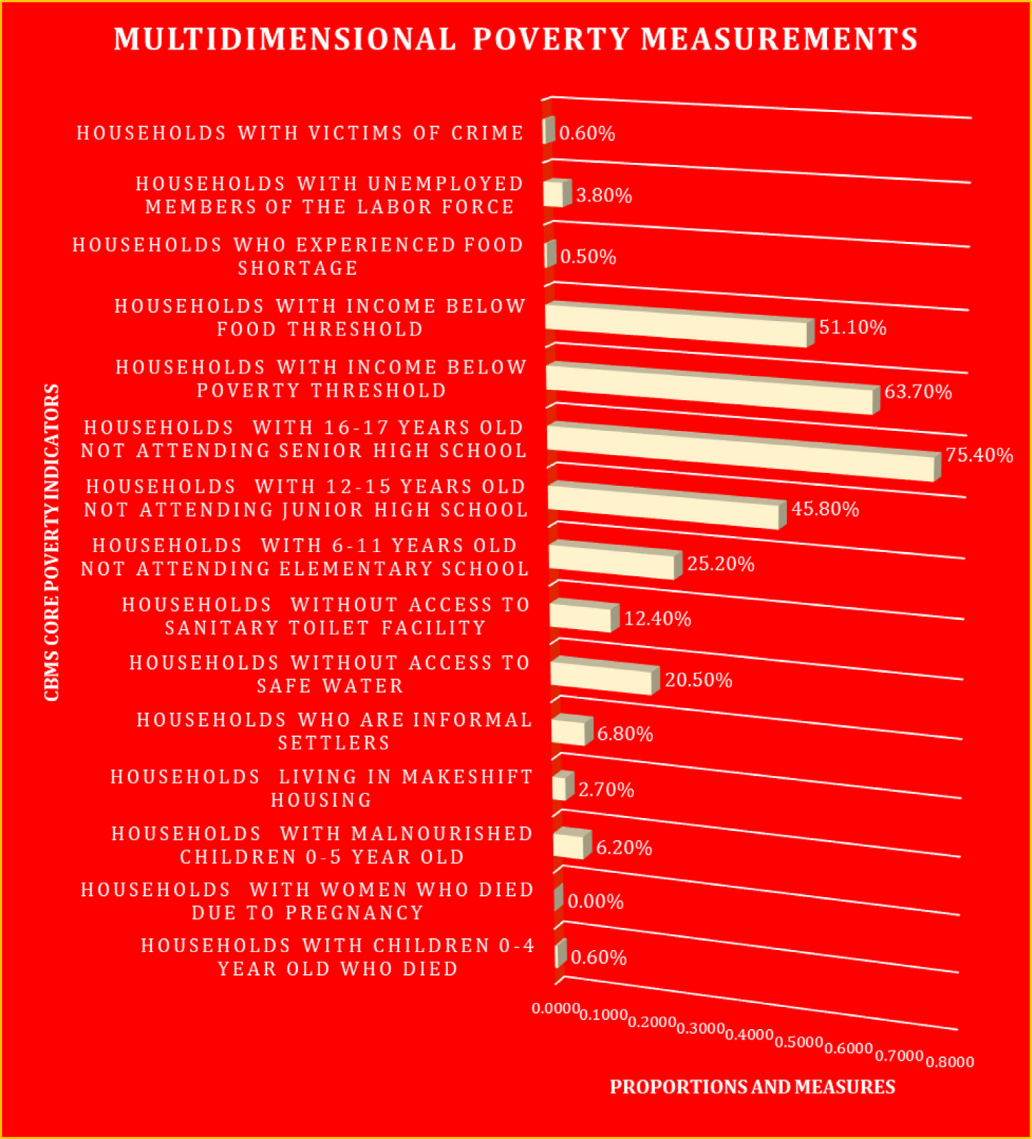
Multidimensional poverty measurements through the multiple constructs and indicators.

Table 9 reveals various indicators based on constructs on how to measure multidimensional poverty. By utilizing the list of indicators presented in Table 1, Table 2, Table 3, it would be easy for researchers to replicate or reimplement the poverty measures. Through the utilization of data analytics and econometrics tools, a very useful table can be generated to measure the unmeasurable and multidimensional poverty [8,9]. Moreover, Fig. 5 showcases the results of poverty measurements through multiple constructs and indicators. It reflects the summary of Table 7 that can be translated into a chart to better understand the poverty cases in a particular region.

**Table 7:** Intervening variables that can be utilized by the public for economic development.

Intervening Variables	Descriptions
I_Settlers x TNOHH_Members	Households who are informal settlers x Household Members
MSH_Dwellers x C_Mortality	Households living in makeshift housing x Children under 5 years old who died
WAS_Water x β_1_C_Mortality	Households without access to safe water x Children under 5 years old who died
WAS_Water x TNOHH_Members	Households without access to safe water x Household Members
WAS_Water x I_Settlers	Households without access to safe water x Households who are informal settlers
WAS_Water	Households without access to safe water x Households living in makeshift housing
WASF_Toilet x I_Settlers	Households without access to sanitary toilet facility x Households who are informal settlers
WASF_Toilet x MSH_Dwellers	Households without access to sanitary toilet facility x Households living in makeshift housing
I_Settlers x TNOHH_Members	Households who are informal settlers x Household Members
DRRP x MSH_Dwellers	Disaster Risk Preparedness x Makeshift Housing
DRRP x CO_Typhoon	Disaster Risk Preparedness x Child Mortality
DRRP x TNOHH_Members	Disaster Risk Preparedness x Total Household Members
DRRP x C_Mortality	Disaster Risk Preparedness x Calamity Occurrences

**Table 8:** Classification matrix of poverty prediction that can be utilized by the public to derive insights about multidimensional poverty.

Classified	Poor	Non-Poor	Total
	6332	1658	7990
	1840	3264	5104
Total	8172	4922	13094
Sensitivity			77.48%
Specificity			66.31%
Positive predictive value			79.25%
Negative predictive value			63.95%
			
False + rate for true Non-Poor			33.69%
False - rate for true Poor			22.52%
False + rate for classified +			20.75%
False - rate for classified -			36.05%
*Correctly classified*			*73.29*%

Furthermore, Table 10 depicts the results of non-linear (logistic) regression on poverty outcomes that were measured or predicted by different constructs and indicators. Tables 6 and 7 reveal the indicators utilized and models 1 and 2 served as benchmarks for the analysis. x refers to the coefficient of the variables while P reveals the p-values at the four disaggregated configurations (4 sectors = Isarog, Poblacion, Salog, and Ranggas) and then the combined levels at Goa, municipality.

**Table 9:** Measuring multidimensional poverty through multiple constructs and indicators.

爱耻		挨		宅	
		HHM	HHP	MEMM	MEMP
诶	α * Ξ * ξ	α	Α = α / Ξ	α	Α = α / ξ
	β	β	Β = β / H	β	Β = β / N
	γ * Ι * ι	γ	Γ = γ / Ι	γ	Γ = γ / ι
必	δ	δ	Δ = δ / H	δ	Δ = δ / N
	ε	ε	Ε = ε / H	ε	Ε = ε / N
西	ζ	ζ	Ζ = ζ / H	ζ	Ζ = ζ / N
	η	η	S = η / H	η	S = η / N
衣	θ * Κ * κ	θ	Θ = θ / Κ	θ	Θ = θ / κ
	λ * Ο * ο	λ	Λ = λ / Ο	λ	Λ = λ / ο
	μ * π * Π	μ	Μ = μ / π	μ	Μ =μ / Π
艾付	ρ	ρ	Ρ = ρ / H	ρ	Ρ = ρ / N
	φ	φ	Φ = φ / H	φ	Φ = φ / N
	χ	χ	Χ = χ / H	χ	Χ = χ / N
	Ψ * Υ * υ	Ψ	Ψ = Ψ / Υ	Ψ	Ψ = Ψ / υ
记	ω	ω	Ω = ω / H	ω	Ω = ω / N

**Table 10:** Results of non-linear regression on the multidimensional poverty decomposition with intervening combination at various configurations.

Poverty Outcomes	Isarog		Poblacion		Salog		Ranggas		Goa	
	x	P	x	p	x	p	x	p	x	p
C_Mortality	0.23	0.00	1.13	0.05	0.91	0.04	0.94	0.27	0.98	0.05
M_Mortality	0.00	0.00	0.00	0.00	0.00	0.00	0.00	0..00	0.00	0.00
C_Malnutrition	0.78	0.05	0.96	0.03	0.93	0.03	1.89	0.00	1.29	0.00
I_Settlers	0.62	0.04	0.81	0.04	0.90	0.04	0.11	0.61	0.44	0.00
MSH_Dwellers	0.34	0.00	0.30	0.01	0.25	0.02	0.35	0.04	0.47	0.03
WAS_Water	0.02	0.02	0.59	0.00	0.35	0.00	0.41	0.00	0.54	0.00
WASF_Toilet	0.74	0.00	0.80	0.05	-0.62	0.04	0.63	0.00	0.83	0.00
TNOHH_Members	0.28	0.00	0.18	0.00	0.23	0.00	0.27	0.00	0.18	0.00
CNA_Elem	0.21	0.01	0.39	0.00	0.67	0.00	0.67	0.00	0.59	0.00
CNA_JunHS	0.37	0.02	0.47	0.00	0.42	0.04	0.74	0.00	0.61	0.00
CNA_SenHS	0.32	0.03	0.25	0.00	0.53	0.57	0.23	0.09	0.21	0.00
LF_Unmployed	0.14	0.02	0.45	0.05	0.57	0.04	0.16	0.49	0.17	0.05
Vic_Crime	0.03	0.05	0.48	0.04	0.14	0.93	1.37	0.21	0.12	0.02
DR_Prepared	-0.23	0.00	-0.07	0.04	-0.56	1.00	-1.36	0.00	-1.58	0.00
CO_Typhoon	1.79	0.00	0.09	0.00	1.43	1.00	0.00	0.62	0.49	0.00
CO_Flood	0.06	0.01	0.02	0.01	0.21	0.05	-0.19	0.36	0.11	0.05
CO_Drought	3.13	0.00	0.06	0.00	1.11	0.02	0.00	0.05	2.58	0.00
CO_VolEruption	0.00	0.00	0.00	0.00	0.00	0.00	0.00	0.00	0.00	0.00
CO_LMSlide	1.35	0.01	0.02	0.00	1.06	1.00	0.00	0.27	0.87	0.00
I_Settlers x TNOHH_Members	0.08	0.50	0.16	0.01	0.05	0.66	0.03	0.76	0.15	0.02
MSH_Dwellers X C_Mortality	0.10	0.04	0.04	0.12	0.18	0.12	0.13	0.05	0.08	0.00
WAS_Water x C_Mortality	1.02	0.03	1.68	0.03	0.97	0.22	0.12	0.00	0.32	0.05
WAS_Water x TNOHH_Members	0.05	0.02	0.13	0.02	0.19	0.03	0.07	0.19	0.08	0.01
WAS_Water x I_Settlers	1.38	0.01	1.80	0.01	1.40	0.05	0.45	0.35	0.14	0.06
WAS_Water x MSH_Dwellers	0.58	0.04	0.91	0.04	0.24	0.04	0.47	0.47	0.22	0.07
WASF_Toilet x I_Settlers	0.34	0.05	0.96	0.05	0.02	0.06	0.87	0.21	0.13	0.04
WASF_Toilet x MSH_Dwellers	0.12	0.89	2.71	0.67	3.23	0.03	0.95	0.18	1.21	0.33
DR_Prepared x MSH_Dwellers	-0.18	0.02	-1.24	0.02	-0.39	0.02	-0.83	0.09	-0.63	0.00
DR_Prepared x C_Mortality	-0.20	0.12	-0.08	0.20	-0.21	0.20	-0.32	0.00	-0.05	0.00
DR_Prepared x TNOHH_Members	-0.25	0.00	-0.38	0.00	1-.57	0.44	-0.89	0.22	-0.27	0.02
DR_Prepared x CO_Typhoon	.-.88	0.02	-0.23	0.03	0.13	0.39	-0.45	0.00	-0.66	0.00
Constant	-2.22	0.00	-1.00	0.00	-1.32	0.00	-0.32	0.15	2.31	0.00

*Note:* x = coefficients and P = p-values

## Limitations

The analyzed data provided in this work has no known limitation because it utilizes a complete enumeration of all the households and populations. Therefore, it is not exposed to any sampling error, thus satisfying the internal and external validity of sample assumptions. The data covers multidimensional variables that are carefully analyzed and filtered [1]. The dataset has no known biases nor limited quality size. However, time restrictions are present. Generating reliable and verifiable datasets takes time. The data covers 2018–2020 conditions and the next data update takes time to occur.

## Ethics Statement

Approval for conducting this study was granted by the Partido State University and the University of the Philippines Los Baños. Approval for utilizing, cleaning, analyzing, and disseminating processed data was granted by the Local Government Unit of Goa, Camarines Sur. This was a voluntary, and non-experimental data analysis. The involvement of human subjects in this work is indirect and secondary. Hence, no ethical clearance applies. The current work also does not involve direct human subjects, animal experiments, or any data collected from social media platforms.

## CRediT Author Statement

**Emmanuel Onsay:** Conceptualization, Original draft preparation, Data Curation, Writing, Methodology, Econometric Modeling, Economic Analysis, Validity tests, and Editing. **Jomar F. Rabajante:** Conceptualization, Supervision, Software, Data analytics modeling, mathematical analysis, Validation, Writing, Reviewing and Editing.

## Data Availability

Dataset on Measuring the Unmeasurable Multidimensional Rural Poverty for Economic Development: Analysis from the Poorest District of the Poorest Province in the Poorest Region of Luzon, Philippines (Original data) (Mendeley Data).
